# Age-Dependent Clinical Relevance of Lipoprotein(a): A Comprehensive Review from Childhood to Adulthood

**DOI:** 10.3390/jcm14176018

**Published:** 2025-08-26

**Authors:** Athina Nasoufidou, Agni Glava, Maria Mavridou, Panagiotis Stachteas, Efstratios Karagiannidis, Dimitrios Patoulias, George Kassimis, Nikolaos Fragakis, Maria Kavga

**Affiliations:** 1 Second Cardiology Department, Medical School, Hippokration General Hospital, Aristotle University of Thessaloniki, 54124 Thessaloniki, Greecestratoskarag@gmail.com (E.K.); gksup@yahoo.com (G.K.); nfrag@auth.gr (N.F.); 2 4th Department of Pediatrics, Papageorgiou General Hospital, School of Medicine, Faculty of Health Sciences, Aristotle University of Thessaloniki, Ring Road, Municipality of Pavlou Mela, Area N. Evkarpia, 56429 Thessaloniki, Greece; agnes.glava@gmail.com; 3 2nd Propedeutic Department of Internal Medicine, Faculty of Medicine, School of Health Sciences, Aristotle University of Thessaloniki, 54124 Thessaloniki, Greece; mmavrida@yahoo.gr (M.M.); dipatoulias@gmail.com (D.P.); 4 Department of Emergency Medicine, AHEPA University Hospital, 54636 Thessaloniki, Greece; 5 3rd Department of Pediatrics, School of Medicine, Hippokration General Hospital, Aristotle University of Thessaloniki, 54642 Thessaloniki, Greece; mkavga@auth.gr

**Keywords:** lipoprotein a, cardiovascular disease, childhood, adulthood, atherosclerosis, familial hypercholesterolemia

## Abstract

Lipoprotein(a) [Lp(a)] has emerged as a significant independent risk factor for atherosclerotic cardiovascular disease (ASCVD). While plasma Lp(a) levels remain relatively stable throughout life, their clinical impact varies depending on age and concentration. This comprehensive review examines the age-dependent clinical relevance of Lp(a), from childhood through adulthood. In pediatric populations, elevated Lp(a) levels are associated with early indicators of vascular dysfunction and with conditions like familial hypercholesterolemia (FH). In adults, elevated Lp(a) is consistently linked to an increased risk of myocardial infarction (MI), stroke, and calcific aortic valve disease, particularly in those with additional cardiovascular risk factors. We also discuss emerging therapies targeting Lp(a) that may significantly alter long-term cardiovascular risk if implemented early. Understanding the lifelong implications of elevated Lp(a) highlights the need for age-specific strategies for screening, monitoring, and intervention. Future research should prioritize identifying high-risk pediatric populations, refining risk thresholds, and determining optimal timing for therapeutic initiation to improve long-term cardiovascular outcomes.

## 1. Introduction

Lp(a), predominantly genetically determined, has been described as a risk factor for CVD across all age groups, with values <30 mg/dL, ≈75 nmol/L generally considered normal. Lp(a) has been investigated in young adults with no traditional cardiovascular risk factors, and even in children who experienced cardiovascular events such as stroke [1]. In some of these cases, Lp(a) was found to be elevated, identifying it as a possible risk factor. With further investigation, it was confirmed that Lp(a) either contributes directly to CVD or exists as a residual cardiovascular risk factor for future events [2]. Elevated Lp(a) levels have been associated with a range of specific outcomes, including myocardial infarction, angina pectoris/coronary stenosis, ischemic stroke, carotid stenosis, peripheral arterial disease, aortic valve stenosis, heart failure, cardiovascular mortality, all-cause mortality, and possibly venous thromboembolism [2]. Lp(a) is particularly important in individuals with a family history of premature ASCVD and FH, as emphasized in current clinical guidelines [3]. The measurement of Lp(a) in these populations supports earlier identification of at-risk individuals. It may guide the implementation of more aggressive lifestyle modifications and, eventually, targeted pharmacologic interventions once effective Lp(a)-lowering therapies become available.

## 2. Biology and Genetics of Lipoprotein(a)

Lp(a) is structurally similar to low-density lipoprotein (LDL) in terms of size, lipid content, and the presence of apolipoprotein B100 (apoB). The key distinguishing feature of Lp(a) is the addition of apolipoprotein(a) [apo(a)], which is another protein covalently linked to apoB via a disulfide bond. Apo(a) shares remarkable structural similarity with plasminogen, with approximately 94% amino acid sequence homology. Apo(a) contains an enzymatically inactive protease domain and several kringle domains—looped protein structures named for their resemblance to Scandinavian pastries. Two main kringle types are present: Kringle V (KV), found as a single copy, identical in sequence to plasminogen by 91%, and Kringle IV (KIV). The KIV domain can appear in 10 distinct subforms (KIV types 1–10). Among these, KIV type 2 is highly repetitive and can appear 10 to 40 times within apo(a). The number of KIV type 2 repeats is genetically determined and highly variable among individuals, ranging from 12 to 51 copies. This variability results in at least 34 different apo(a) isoforms, influencing the size of the Lp(a) particle and its plasma concentration, with smaller isoforms generally associated with higher Lp(a) levels and increased cardiovascular risk [4].

In addition to the KIV repeat polymorphisms, copy number variations (CNVs) within the LPA gene also influence Lp(a) concentrations [5]. CNVs are duplications or deletions in the LPA gene that can decrease or increase the Lp(a) concentrations. The 1-copy LPA gene variant is protective for CVD, for example, in a Chinese case-control study [6].

Another important factor influencing total plasma Lp(a) levels is the presence of single-nucleotide polymorphisms (SNPs) in the LPA gene. Over 2.000 SNPs have been linked to Lp(a) concentrations, but two in particular—rs10455872 and rs3798220—have emerged as the most strongly associated. These SNPs do not directly alter the production of Lp(a), but they are closely linked to the presence of small apo(a) isoforms, which are known to result in higher plasma Lp(a) levels. In fact, approximately half of individuals carrying small apo(a) isoforms also carry one of these genetic variants. Due to their strong predictive value for elevated Lp(a) and associated cardiovascular risk, these SNPs have become key targets in genomic research [7]. SNPs have also been found to strongly participate in valvular heart disease, especially aortic stenosis [8].

Lipoprotein(a) [Lp(a)] promotes inflammation through multiple interconnected mechanisms. By binding oxidized phospholipids, Lp(a) facilitates the differentiation of monocytes into macrophages and enhances their migration and adhesion to the endothelium. It also stimulates the secretion of chemokines, reactive oxygen species (ROS), and matrix metalloproteinase-9 (MMP-9), which contribute to the destabilization of atherosclerotic plaques [9]. Additionally, Lp(a) activates inflammatory pathways such as NF-κB via receptors including TLR2, TLR4, CD14, and CD36, resulting in increased production of pro-inflammatory cytokines like IL-8, IL-1β, and TNF-α. The resulting inflammatory cytokines, ROS, and adhesion molecules (VCAM-1, ICAM-1, and E-selectins) disrupt endothelial cell junctions and cytoskeletal integrity, promoting endothelial autophagy and metabolic shifts such as enhanced glycolysis, thereby exacerbating vascular inflammation [10]. Furthermore, Lp(a) inhibits the activation of transforming growth factor-beta (TGF-β) and upregulates platelet-derived growth factor (PDGF), stimulating smooth muscle cell (SMC) proliferation and sustaining the inflammatory environment within the vessel wall [11]. Together, these processes create a chronic pro-inflammatory state that plays a crucial role in the progression of atherosclerosis and cardiovascular disease.

## 3. Lp(a) Expression and Measurement Over the Lifespan

### 3.1. Children and Adolescents

Lp(a) levels are low at birth, with the Lp(a) gene primarily becoming active and expressed by the age of two. These levels continue to fluctuate and stabilize, reaching adult concentrations around five years of age. After this point, Lp(a) levels remain relatively stable throughout life. Approximately 20% of children have increased Lp(a) in childhood, and 17% of them do not have increased LDL-C levels [12]. Lp(a) levels are independent of age, sex, and body weight across childhood [13].

### 3.2. Adults and Elders

Lp(a) levels remain relatively stable over time in adulthood and are minimally influenced by lifestyle factors such as diet and exercise or most medications, including statins [14]. Exceptions may occur in specific clinical contexts, such as chronic kidney disease or certain endocrine disorders. In the general adult population, Lp(a) levels greater than 50 mg/dL (approximately 125 nmol/L) are considered elevated and are associated with an increased risk of ASCVD. Approximately 28% of individuals have Lp(a) levels above this threshold, while around 10% have levels exceeding 100 mg/dL (≈250 nmol/L) [2].

### 3.3. Population Variations

Lp(a) levels and isoform distributions vary significantly among ethnic groups, largely due to genetic differences. African populations typically have higher Lp(a) levels, but their isoform patterns differ from those of other groups. Europeans are more commonly carriers of specific SNPs (such as rs10455872 and rs3798220) that significantly influence Lp(a) concentrations [15]. In contrast, Asian populations tend to have lower Lp(a) levels and distinct isoform profiles. These variations underscore the fact that genetic factors account for over 90% of the variability in Lp(a) levels across individuals and populations [2].

Special consideration should be given to sex-related differences that may influence Lp(a) levels. Endogenous sex hormones appear to have minimal to no impact on Lp(a) concentrations. However, physiological states such as pregnancy can significantly elevate Lp(a) levels, in some cases nearly doubling them. Menopause itself does not substantially alter Lp(a) levels, but postmenopausal hormone replacement therapy has been shown to reduce Lp(a) by approximately 25% [14]. Additionally, chronic kidney disease and thyroid dysfunction can influence Lp(a) levels, highlighting the importance of evaluating and managing underlying endocrine conditions when interpreting Lp(a) values [2].

## 4. Risk-Stratification According to Age

### 4.1. Children and Adolescents

Lp(a) has been extensively studied for its role in influencing cardiovascular risk and contributing to premature cardiovascular events, particularly stroke, early in life. The historically significant Bogalusa Heart Study was among the first to highlight the role of Lp(a) in the pathogenesis of atherosclerosis. In a cohort of 2438 children aged 8–17 years, Lp(a) levels were found to be significantly higher in those with a positive family history of MI compared to those without (22.4 vs. 17.1 mg/dL). This association was even stronger among children with Lp(a) levels above 25 mg/dL. The authors concluded that Lp(a) measurement is crucial for early coronary artery disease (CAD) risk assessment beginning in childhood [16]. Numerous other studies and meta-analyses in recent years have consistently supported the role of elevated Lp(a) as a risk factor for premature atherosclerosis starting early in life [17,18,19,20]. Zawacki et al. notably found that Lp(a) was a stronger predictor of premature CVD than LDL-C in relatives of children with FH [21]. This was further confirmed in a cohort of 700 pediatric patients with FH from the LIPIGEN study, which demonstrated a clear association between both high Lp(a) and LDL-c levels with early-onset CVD [22]. Additionally, results from the Cardiovascular Risk in Young Finns Study have shown that elevated Lp(a) levels in youth are linked to an increased risk of major cardiovascular events later in adulthood [23].

Moreover, early signs of vascular dysfunction and atherosclerosis may be detected in children with elevated Lp(a), especially those with FH. As early as 1998, a small case-control study reported impaired flow-mediated dilation (FMD) in children with FH compared to healthy peers [24]. In 2015, another study found an inverse relationship between Lp(a) levels and FMD in 11-year-old children, suggesting early vascular impairment. Notably, this association was also seen in children who received dietary counseling from early life [25]. Furthermore, a 20-year randomized controlled trial involving 200 children with FH showed that elevated Lp(a) levels were linked to increased carotid intima–media thickness (IMT) over time, supporting the value of early Lp(a) screening [26]. However, findings are controversial. A recent retrospective study of 113 children aged 6–18 found no relationship between elevated Lp(a) and IMT, even when LDL was also high [27]. Similarly, the Young Finns study, which measured Lp(a) at ages 17 and 38, found no association between Lp(a) and progression of atherosclerosis as assessed by IMT and FMD [28]. Additionally, a more recent study in young adults with FH found no correlation between Lp(a) and arterial stiffness measured by carotid pulse wave velocity (PWV) [29]. A study from Greece involving 27 children with elevated Lp(a) levels around the age of ten compared to age-matched controls assessed vascular function using carotid IMT, PWV, augmentation index (AIx), and subendocardial viability ratio (SEVR). The study found no significant differences in these vascular indices between the two groups [30].

Interestingly, elevated Lp(a) has been associated with hyperinsulinemia and insulin resistance, even in healthy, normal-weight prepubertal children [31]. Additionally, a study from Greece reported higher spexin levels—a peptide hormone involved in lipid metabolism, adiposity, and appetite regulation—in post-menarcheal adolescent females with elevated Lp(a) [32]. Emerging research has also linked Lp(a) to other early-life factors. For instance, a study examining vascular function in children conceived through assisted reproductive technologies (ART) found significantly higher Lp(a) levels in the ART group compared to spontaneously conceived peers, suggesting potential long-term cardiovascular implications [33]. Moreover, elevated Lp(a) levels have been associated with low birth weight, indicating a possible role of prenatal factors in Lp(a) regulation [34].

### 4.2. Adults

In adults, studies have clearly and consistently associated high Lp(a) with increased risk for MI, coronary death, and ischemic stroke [35,36,37,38]. Specifically, in patients with acute MI, persistently elevated Lp(a) levels were associated with a significantly increased risk of major adverse cardiovascular and cerebrovascular events over a 50-month follow-up period [39]. In one study, Lp(a) levels > 150 mg/dL were linked to higher rates of ASCVD events in both individuals with and without prior cardiovascular history [40]. In a sub-analysis of the PROMISE trial, Lp(a) levels > 50 mg/dL were associated with an increased risk of obstructive CAD, independent of LDL cholesterol levels. However, this association did not extend to high-risk plaque features when analyses were limited to cases with ≥50 or ≥70% coronary stenosis [41]. Additionally, elevated Lp(a) has been associated with coronary artery calcification, as demonstrated by coronary computed tomography imaging [42]. In the ATTICA study, Lp(a) levels were associated with a higher incidence of ASCVD over a 20-year follow-up in unadjusted models [43]. Notably, the added risk conferred by elevated Lp(a) on top of traditional cardiovascular risk factors was quantified as a 68%, 41%, and 14% increase in individuals at low, intermediate, and high baseline risk, respectively [7]. Lp(a) has also been investigated as a marker for subclinical atherosclerosis. In a recent Chinese study involving a general health check-up population, elevated Lp(a) levels were significantly associated with increased carotid intima-media thickness, the presence of carotid plaques, subclinical brain infarcts, and coronary artery calcification [44]. Beyond atherosclerosis, Lp(a) has also been implicated in aortic valve stenosis, with significantly higher levels observed in affected patients [45]. Emerging evidence suggests associations between elevated Lp(a) and other cardiovascular morbidities, including heart failure [46,47,48]—potentially due to coexisting CAD—and atrial fibrillation [49], possibly mediated by atrial structural remodeling.

### 4.3. Elders

The association between Lp(a) levels and cardiovascular events in older adults remains less well-defined, as traditional risk factors often lose predictive power in this population. However, emerging evidence suggests that elevated Lp(a) continues to be a significant and independent risk factor for adverse cardiovascular outcomes in the elderly [50]. In a prospective study involving 5888 patients, increased Lp(a) levels were found to be an independent predictor of stroke, vascular death, and death by any cause in older men, but this association was observed in women [51]. Ιn another study, patients aged ≥ 80 years old with ST elevation MI, those with Lp(a) > 30 mg/dL had 1.5-fold higher risk of cardiovascular death compared to those with Lp(a) ≤ 10 mg/dL, with no major sex differences reported [52]. Similarly, a U.S. prospective cohort study showed that patients over 70 years had an increased absolute incidence of acute coronary syndromes over an 8-year follow-up period [53].

In elderly men hospitalized with chronic heart failure, high Lp(a) levels were associated with the presence of cardio-renal syndrome, further complicating their clinical course [53]. Another important finding comes from a study of older adults without prior CVD: individuals in the highest quartile for both LDL cholesterol (>4.90 mmol/L) and Lp(a) (>276 mg/L) had nearly double the risk of developing coronary heart disease compared to those with lower levels. Interestingly, the study concluded that in elderly individuals with low Lp(a), elevated LDL might not necessitate aggressive lipid-lowering therapy, suggesting a potential role for Lp(a) in guiding treatment decisions [54,55]. Additionally, Lp(a) has been implicated in the pathogenesis and severity of aortic valve stenosis (AVS) among older adults. Those with elevated Lp(a) tend to have more advanced AVS compared to their healthy peers [56]. Elevated Lp(a) levels have also been linked to the occurrence of first-time ischemic or non-embolic stroke in older patients, independently of other cardiovascular risk factors [57].

**Figure 1:**
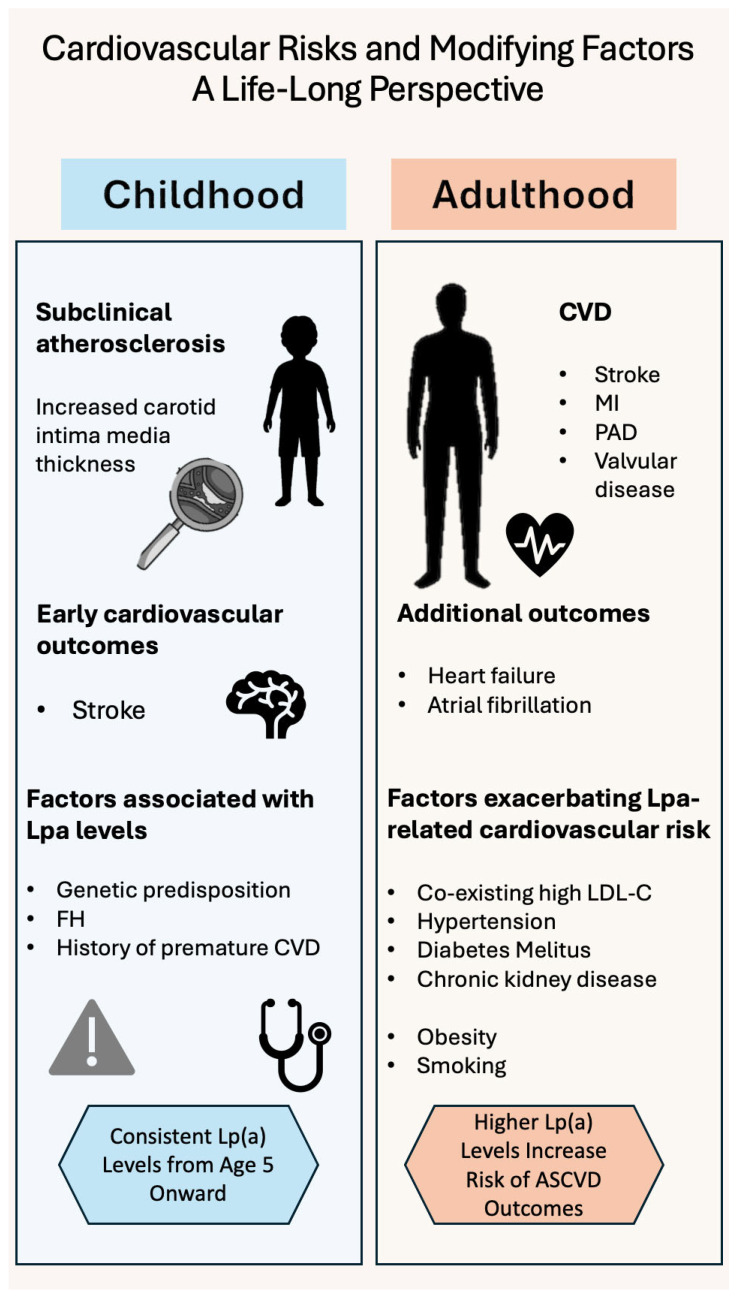
Cardiovascular risks and modifying factors of Lp(a) in a life-long perspective. FH: familial hypercholesterolemia; CVD: cardiovascular disease; ΜΙ: myocardial infarction; PAD: peripheral artery disease; LDL-C: low-density lipoprotein C; ASCVD: atherosclerotic cardiovascular disease.

Table 1 summarizes the main studies according to age group and cardiovascular event, and Figure 1 summarizes the cardiovascular risks and modifying factors across the life span.

## 5. Screening

### 5.1. Children and Adolescents

Measurement of Lp(a) in children is not recommended as part of routine clinical assessments. However, targeted screening may be appropriate in children with a family history of FH, elevated Lp(a), or premature ASCVD. In such cases, early identification of elevated Lp(a) can help recognize individuals at lifelong increased cardiovascular risk [58].

### 5.2. Adults and Elders

Current guidelines recommend measuring Lp(a) once in adulthood to identify individuals with inherited high Lp(a) and increased risk for CVD, as they have heterozygous FH, or to re-evaluate those with intermediate to high risk [2]. This recommendation is based on the genetic determination and relative stability of Lp(a) levels throughout adulthood, making a single measurement sufficient for risk stratification [59].

Current cardiovascular risk calculators, such as SCORE2 [60] and the ASCVD Risk Estimator [61], do not incorporate Lp(a) in their formal risk scoring algorithms, because established treatment thresholds and clinical outcome data following Lp(a) reduction are lacking. Instead, they rely on traditional risk factors such as age, sex, blood pressure, cholesterol levels, smoking status, and diabetes. Although Lp(a) is not yet formally included in standard risk prediction models, its increasingly recognized role in cardiovascular pathology, along with the development of targeted Lp(a)-lowering therapies, may lead to greater clinical emphasis and potential inclusion in future versions of these calculators. This advancement could improve risk stratification by identifying individuals currently classified as low-risk who actually have an elevated cardiovascular risk [2].

## 6. Therapy

### 6.1. Lifestyle Modifications and Current Pharmacological Options

Currently, there is no approved treatment specifically targeting elevated Lp(a) in either children or adults. Most management strategies are derived from adult data and focus primarily on reducing overall cardiovascular risk rather than lowering Lp(a) itself [62].

Lifestyle modifications—including maintaining a healthy diet, engaging in regular physical activity, and avoiding smoking—are universally recommended [63]. While these measures have minimal direct effect on Lp(a) concentrations, they help reduce the burden of other cardiovascular risk factors such as hypertension, obesity, and diabetes. Family counseling is also an essential component of care [63].

Lipid-lowering therapies commonly used in children with FH and adults, such as statins and ezetimibe, do not significantly reduce Lp(a) levels [64]. Similarly, monoclonal antibody proprotein convertase subtilisin/kexin type 9 inhibitors (PCSK9is), which are sometimes prescribed in pediatric FH or adults, have only modest or inconsistent effects on Lp(a) [65,66,67,68]. For individuals with extremely elevated Lp(a) and progressive CVD despite maximal medical therapy, lipoprotein apheresis has been used only in a few specialized centers [69]. This extracorporeal procedure can reduce Lp(a) concentrations by more than 70%, but it is invasive, costly, requires frequent sessions, and is only available in selected institutions [70].

Given these limitations—namely, the absence of specific guidelines and effective treatments—attention has shifted to novel Lp(a)-targeted therapies currently under investigation. These agents show promising results, with potential reductions in Lp(a) levels exceeding 70% [71]. However, emerging data suggest that to achieve a meaningful reduction in cardiovascular risk, an absolute Lp(a) decrease of over 100 mg/dL may be required—equivalent in clinical benefit to a 40% reduction in LDL [72]. Currently, there is no conclusive evidence that these therapies improve cardiovascular outcomes, and such confirmation will require the completion of ongoing or future phase III trials. Emerging therapies are in advanced clinical trials in adults but are not yet studied or approved for pediatric use.

### 6.2. Novel Treatments

The most promising results in Lp(a) reduction come from novel RNA-based therapies, particularly antisense oligonucleotides (ASOs) and small interfering RNAs (siRNAs). These therapies target the production of apo(a), a key component of Lp(a) particles [73].

### 6.3. Antisense Oligonucleotides (ASOs)

ASOs work by binding to the complementary apo(a) mRNA sequence, promoting degradation via RNase H1, an intracellular enzyme that recognizes and cleaves RNA-DNA hybrids. The most advanced ASO is pelacarsen, which, in a phase II trial involving 288 patients with established CVD, reduced Lp(a) levels by up to 80% with a 20 mg subcutaneous dose administered every 4 weeks [74]. Pelacarsen is now being evaluated in a large phase III randomized controlled trial to determine whether Lp(a) lowering translates into reduced cardiovascular events [75].

### 6.4. Small Interfering RNAs (siRNAs)

siRNAs bind within cells with the RNA-induced silencing complex (RISC). After removing the sense strand, RISC binds to the antisense strand, which guides the complex to the target mRNA, resulting in its degradation and inhibition of protein synthesis. This mechanism allows for sustained effects with infrequent dosing [76].

Olpasiran has demonstrated Lp(a) reductions of 70–90%, sustained for over six months [77].

Lepodisiran, another siRNA agent, achieved a 94% Lp(a) reduction in a phase II trial and maintained this reduction for over a year after a single repeated dose [78]. Zerlasiran, in a phase II trial of 178 patients with established CVD, reduced Lp(a) by over 90%, with levels remaining 80–85% lower even at week 60 [79]. Zerlasiran is currently being tested in a phase III trial—the first to include individuals without established CVD, assessing its role in primary prevention [80]. All aforementioned RNA therapies are subcutaneous therapies.

### 6.5. Oral Therapy—Muvalaplin

Muvalaplin is a novel oral small molecule that blocks the noncovalent interaction between apo(a) and apoB, preventing Lp(a) particle formation. It has shown an average 65% reduction in Lp(a) levels. While its clinical benefit remains unproven, the ease of oral administration makes it an attractive candidate for further development [81].

### 6.6. Future Directions—Gene Editing

A potential long-term or even one-time solution could come from genome editing. In a mouse model, delivery of CRISPR-Cas9 via an adeno-associated virus vector successfully disrupted the LPA gene in the liver, nearly eliminating circulating apo(a) within 7 days [82]. While human applications are still in early development, genome editing offers exciting prospects for permanent Lp(a) reduction.

**Figure 2:**
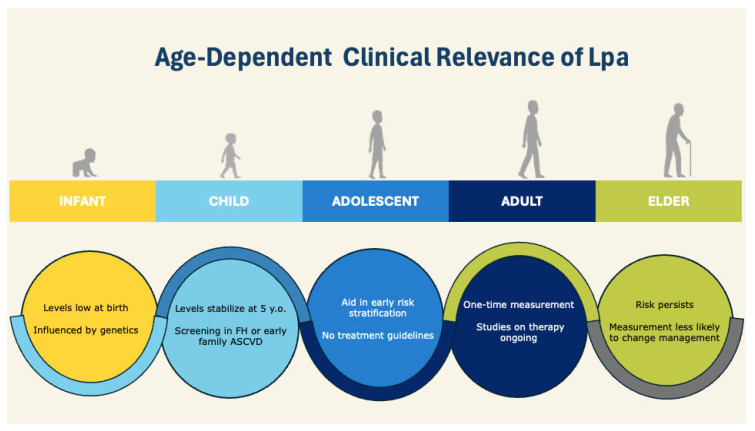
Age-dependent clinical relevance of Lp(a). y.o., years old; FH, familial hypercholesterolemia; ASCVD, atherosclerotic cardiovascular disease.

Figure 2 summarizes key clinical associations and implications of Lp(a) across different age groups.

## 7. Limitations of Current Literature

Despite increasing interest in Lp(a) as a marker for CVD, the majority of studies have focused predominantly on adult populations or specific clinical subgroups, resulting in scarce and fragmented pediatric and elderly data. Longitudinal studies tracking Lp(a) levels and their clinical implications from childhood through adulthood remain limited. Pediatric studies are typically small, observational, and retrospective. Additionally, markers of subclinical atherosclerosis are challenging to study and define due to variability in measurement methods and conflicting results, which complicates the interpretation of outcomes. Furthermore, heterogeneity in study designs, populations, and measured outcomes contributes to inconsistent findings across the literature. Addressing these limitations is essential to advance our understanding of the age-dependent impact of Lp(a) levels.

## 8. Gaps in Evidence—Future Directions

### 8.1. Children and Adolescents

Special considerations apply to all clinical trials involving children, primarily related to safety and ethical concerns, as children are still growing and have different metabolic profiles compared to adults [83]. Therefore, dosing, efficacy, and long-term safety must be thoroughly evaluated before administering treatments to pediatric patients [84].

Moreover, pediatric clinical trials must carefully select appropriate endpoints, emphasizing early markers of atherosclerosis and vascular health to assess treatment efficacy in the absence of immediate clinical events [70].

Successful development and implementation of such trials depend on close collaboration among families, participants, and healthcare providers, with the ultimate goal of extending safe and effective Lp(a)-lowering therapies to children for improved cardiovascular prevention across the lifespan.

Future research should focus on age-specific groups—such as preschool children, when Lp(a) levels stabilize, and adolescents undergoing puberty, when hormonal changes occur—to determine whether early intervention can effectively reduce cardiovascular risk and future events. However, it is important to consider that children may require prolonged or lifelong treatment, which could present challenges in adherence, require family consent, and potentially impact long-term outcomes [85].

### 8.2. Elders

Most current evidence derives primarily from studies focused on younger or middle-aged cohorts, resulting in an underrepresentation of individuals over 70 years of age. This limits the ability to define age-specific thresholds or guide therapeutic strategies and underscores the need for dedicated studies in this population.

## 9. Discussion

The conception that Lp(a) is a major inherited cardiovascular risk factor has progressively advanced over the years, and evidence consistently demonstrates its association with heightened risk for ASCVD [2]. While most research has focused on adults, growing evidence in pediatric patients suggests that high Lp(a) levels established in early childhood may contribute to subclinical atherosclerosis, especially in genetically predisposed individuals with FH [4].

Despite the strong observational associations, a major limitation remains the lack of interventional data showing that lowering Lp(a) improves clinical outcomes. This is partly because there has been no specific therapy for elevated Lp(a), as current studies are still ongoing. Traditional lipid-lowering therapies have little or no effect on Lp(a), and lipoprotein apheresis, though effective, is invasive and limited in availability [69].

Studies on Lp(a) should also consider its heterogeneity across ethnic groups, age ranges, and comorbidities [42]. Pediatric research in this area poses additional challenges for many reasons, and early surrogate markers of vascular dysfunction may be difficult to assess reliably [40]. Therefore, there is a clear need for longitudinal studies starting in childhood and extending into adult life to fully understand the long-term vascular impact of elevated Lp(a). However, such studies are very difficult to carry out in practice.

Screening recommendations remain conservative in children, with targeted measurements advised only when FH or a family history of premature ASCVD is present, and a one-time measurement is typically recommended in adulthood [85]. Lp(a) is not yet incorporated into cardiovascular risk calculators, but this will likely change once effective treatments become available.

Briefly, in clinical practice, a single Lp(a) measurement after age 5 can identify individuals at genetically elevated risk for ASCVD, allowing optimization of other modifiable risk factors even in the absence of specific therapies. In adults and older individuals, a one-time measurement aids in cardiovascular risk assessment and highlights those who may benefit from closer follow-up. With novel Lp(a)-lowering therapies advancing through clinical trials, early detection will be key to targeted prevention and treatment in the near future.

**Figure 3:**
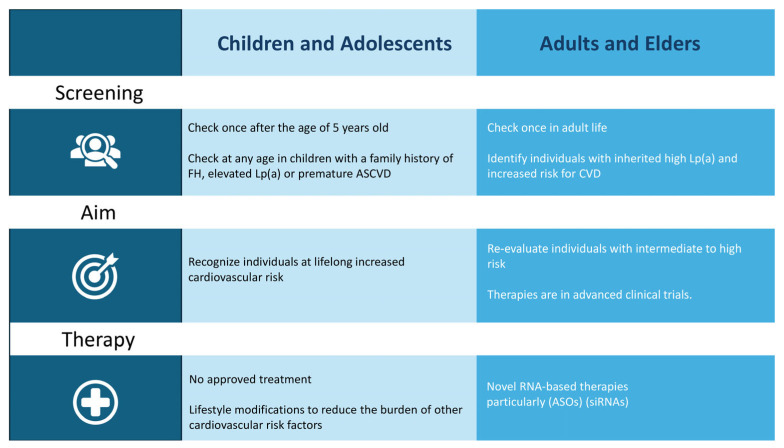
Proposed algorithm for Lp(a) screening and management across the lifespan. FH; familial hypercholesterolemia, ASCVD; atherosclerotic cardiovascular disease, CVD; cardiovascular disease; ASOs; antisense oligonucleotides, siRNAs; small interfering RNAs.

In Figure 3, we present a proposed algorithm for screening and managing Lp(a) levels across different stages of life.

## 10. Conclusions

In conclusion, Lp(a) is a predominantly genetically determined, lifelong risk factor for ASCVD, with substantial evidence supporting its role in the pathogenesis of atherosclerosis from childhood onward. Elevated Lp(a) levels, particularly in individuals with FH or a family history of premature ASCVD, contribute to early vascular changes and increased long-term CVD risk. Although routine screening and treatment have been limited by the absence of specific therapies, the landscape is rapidly evolving. Recent advances in RNA-based therapies, such as ASOs and si-RNAs, have demonstrated the ability to reduce Lp(a) concentrations by over 70%, providing a solid basis for future clinical research. As novel targeted agents advance through clinical studies, there is optimism that clinicians will soon have effective means to mitigate the cardiovascular burden associated with elevated Lp(a). To fully realize the potential benefits of Lp(a) lowering, further longitudinal studies are essential, focusing on. Future research should continue to refine screening strategies and determine the clinical benefits of targeted Lp(a) lowering interventions in both pediatric and adult populations.

## Figures and Tables

**Table 1:** Summary of main studies correlating age-specific associations between elevated lipoprotein(a) and cardiovascular events.

Age	Stroke	MI	CAD	Atherosclerosis
**Child**	Bogalusa (*n* = 1587, cohort) [16]		Bogalusa [16] Zawacki et al. (*n* = 129, cohort) [21] LIPIGEN (*n* = 653 *, cohort) [22]	de Boer LM et al. (*n* = 214, cohort) [26] Young Finns (*n* = 2.080, cohort) [28]
**Adolescent**			Zawacki et al. (*n* = 129, cohort) [21] LIPIGEN (*n* = 653 *, cohort) [22]	Young Finns (*n* = 3.596, cohort) [23]
**Adult**	Tipping (*n* = 174.111, meta-analysis) [35]	Tipping (*n* = 102.221, meta-analysis) [35] Wang (*n* = 1.131, cohort) [39] Patel (*n* = 460.506, Biobank) [40]	PROMISE (*n* = 1.185, secondary analysis of an RCT) [41] ATTICA (*n* = 3.042, cohort) [43]	Jackson (*n*= 5.597, cohort) [42]
**Elder**	PROSPER (*n* = =5732, cohort) [50] Ariyo (*n*= 5888, cohort) [51] Milionis (*n* = 328, case-control) [57]	Zhang (*n* = 1.008, cohort) [52] Bartoli (*n* = 755, cohort) [53]	Simons (*n*= 2.805, cohort) [54]	

* Children and adolescents, n: number of participants included in each study; study type is indicated in parentheses.

## Abbreviations

The following abbreviations are used in this manuscript:

Lp(a)	Lipoprotein(a)
ASCVD	Atherosclerotic Cardiovascular Disease
FH	Familial Hypercholesterolemia
LDL	Low-Density Lipoprotein
ApoB	Apolipoprotein B100
ApoA	Apolipoprotein A
KIV	Kringle IV
CNVs	Copy Number Variations
CVD	Cardiovascular Disease
SNPs	Single-Nucleotide Polymorphisms
MI	Myocardial Infarction
CAD	Coronary Artery Disease
FMD	Flow-Mediated Dilation
IMT	Intima-Media Thickness
PWV	Pulse Wave Velocity
SEVR	Subendocardial Viability Ratio
ART	Assisted Reproductive Technologies
AVS	Aortic Valve Stenosis
PCSK9is	Proprotein Convertase Subtilisin/Kexin type 9 Inhibitors
ASOs	Antisense Oligonucleotides
siRNAs	Small Interfering RNAs
RISC	RNA-induced silencing complex
